# Non-instantaneous synaptic transmission in spiking neuron networks and equivalence with delay distribution

**DOI:** 10.1186/1471-2202-14-S1-P267

**Published:** 2013-07-08

**Authors:** Matteo Biggio, Marco Storace, Maurizio Mattia

**Affiliations:** 1 Department of Electrical, Electronic, Telecommunications Engineering, and Naval Architecture, University of Genoa, Genova, Via Opera Pia 11A, 16145, Italy; 2 Department of Technologies and Health, Istituto Superiore di Sanità, Rome, Italy

## 

Synapses in neuron networks filter incoming spikes with a wide variety of time constants, affecting the stability of various collective dynamics [1], the selectivity in transmitting information [2] and the reactivity to suddenly appearing exogenous stimuli [3]. Even for extremely simplified models of spiking neurons and network connectivities, theoretical approaches including non-instantaneous transmission rely on approximations valid for relatively small time scales [3,4] or on the assumption of a quasi-adiabatic dynamical regime [5].

Here, we addressed this issue working out a unified framework in which from small to large synaptic time scales the same approximated theoretical description holds for the firing rate dynamics of spiking neuron networks. Starting from single-neuron stochastic dynamics, we derived a set of ordinary differential equations for the population emission rate relying on the spectral expansion of the associated Fokker-Planck equation, eventually extending to the colored noise case a previous derivation obtained under white noise hypothesis [6]. The resulting population dynamics valid under a low-noise approximation, took into account also finite-size effects. This allowed to compare theoretical power spectra of population emission rates with those estimated from extensive network simulations performed with NEST [7]. Match between theory and simulation was investigated for different integrate-and-fire (IF) neuron models with different levels of complexity, e.g., VLSI IF (VIF [6], a generalization of the Perfect IF model), Leaky IF (LIF), and Exponential IF (EIF), confirming the effectiveness of the reduced theoretical description. Effectiveness which in turn could be further improved relying on higher-order perturbative terms in the fluctuation size of the synaptic current for any synaptic time scale.

Moreover, such perturbative approach allowed to avoid the analytical difficulties of dealing with multi-dimensional Fokker-Planck equations with discontinuous boundary conditions. A simplification which helped in highlighting as main result that networks of neurons with non-instantaneous synapses can, under fairly broad assumptions, be described by equivalent networks with suitable distributions of transmission delays. A formal analogy valid from simple VIF models to more realistic point-like simplifications like LIF or EIF neurons.
